# A novel deep intronic variant strongly associates with Alkaptonuria

**DOI:** 10.1038/s41525-021-00252-2

**Published:** 2021-10-22

**Authors:** Chien-Yi Lai, I-Jung Tsai, Pao-Chin Chiu, David B. Ascher, Yin-Hsiu Chien, Yu-Hsuan Huang, Yi-Lin Lin, Wuh-Liang Hwu, Ni-Chung Lee

**Affiliations:** 1 Department of Medical Genetics, National Taiwan University Hospital, Taipei, Taiwan; 2 Department of Pediatrics, National Taiwan University Children Hospital, Taipei, Taiwan; 3 Department of Pediatrics, National Taiwan University Hospital Hsin-Chu Branch, Hsin-Chu, Taiwan; 4 Department of Pediatrics, Kaohsiung Veterans General Hospital, Kaohsiung, Taiwan; 5 Computational Biology and Clinical Informatics, Baker Heart and Diabetes Institute, Melbourne, VIC Australia; 6 Structural Biology and Bioinformatics, Department of Biochemistry and Pharmacology, University of Melbourne, Melbourne, VIC Australia; 7 Systems and Computational Biology, Bio21 Institute, University of Melbourne, Melbourne, VIC Australia; 8 Department of Biochemistry, Bio21 Institute, University of Cambridge, Cambridge, UK

**Keywords:** Molecular medicine, Metabolic disorders

## Abstract

Alkaptonuria is a rare autosomal recessive inherited disorder of tyrosine metabolism, which causes ochronosis, arthropathy, cardiac valvular calcification, and urolithiasis. The epidemiology of alkaptonuria in East Asia is not clear. In this study, patients diagnosed with alkaptonuria from January 2010 to June 2020 were reviewed. Their clinical and molecular features were further compared with those of patients from other countries. Three patients were found to have alkaptonuria. Mutation analyses of the homogentisate 1,2-dioxygenase gene (*HGD*) showed four novel variants c.16-2063 A > C, p.(Thr196Ile), p.(Gly344AspfsTer25), and p.(Gly362Arg) in six mutated alleles (83.3%). RNA sequencing revealed that c.16-2063 A > C activates a cryptic exon, causing protein truncation p.(Tyr5_Ile6insValTer17). A literature search identified another 6 patients with alkaptonuria in East Asia; including our cases, 13 of the 18 mutated alleles have not been reported elsewhere in the world. Alkaptonuria is rare in Taiwan and East Asia, with *HGD* variants being mostly novel and private.

## Introduction

Human homogentisate 1,2-dioxygenase, a homogentisic acid oxidase encoded by the *HGD* gene, catalyzes the conversion of homogentisic acid (HGA), a metabolite of tyrosine, to 4-maleylacetoacetate. Alkaptonuria (AKU; MIM # 203500) is a rare autosomal recessive disease caused by tissue accumulation and urinary excretion of HGA^1^. The hallmark of the disease is dark urine, which is caused by the oxidization of HGA. Associated morbidities include ochronosis, ochronotic arthropathy, cardiac valvular calcification, and urolithiasis in the 4th to 6th decades of life^2^. Although the life expectancy of patients with AKU is not reduced, their quality of life deteriorates mainly due to painful joint diseases^3^. The disease is not yet curable, and symptomatic relief management, such as physiotherapy, painkillers, and joint replacement therapy, is necessary^4^. Recently, nitisinone, a potent inhibitor of HGA production, was shown to decrease urinary HGA excretion and decelerate disease progression^5^.

The worldwide prevalence of alkaptonuria is estimated to be 1:100,000 to 1:1,000,000^2,6^, but the disease is more prevalent in Slovakia, Jordan, the Dominican Republic, and India^4^. *HGD* p.(Gly161Arg) is a common disease-causing variant in Slovakia and the Czech Republic; *HGD* p.(Cys120Trp) is common in the Dominican Republic^7^. The most prevalent variants in European countries excluding the abovementioned is p.(Met368Val), accounting for 11.2% of all *HGD* variants, followed by p.(Val300Gly), p.(Gly270Arg), and p.(Pro230Ser), which together account for ~9.5%^7,8^. Because AKU is rarely reported in Asia, we conducted this study to examine its characteristics in this region.

## Results

### Demographic data and clinical presentation of patients

Three Taiwanese (Han ethnicity) patients, two males, and one female, with a diagnosis of AKU were identified. Two of the patients had dark urine during infancy or early childhood, and one had dark urine at the age of 13 years. Their current ages are 10–20 years (Table 1). None of them showed a significant elevation in plasma tyrosine.

**Table 1:** Patient clinical presentations and *HGD* variants.

Patient	1	2	3
Age of onset	13 y	Early childhood	1.5 y
Sex	Male	Female	Male
Current age	20 y	12 y	10 y
Variant 1	c.291 G > A p.(Trp97Ter)	c.473 C > T p.(Pro158Leu)	*c.1084 G > A p.(Gly362Arg)
Variant 2	*c.587 C > T p.(Thr196Ile)	*c.1031delG p.(Gly344AspfsTer25)	* c.16-2063 A > C p.Tyr5_Ile6insValTer17
Tyrosine	58.3	91.1	52.7
Ochronosis	-	Sclera	-
Arthralgia	-	Bilateral knees Right hip	Bilateral knees

Tyrosine: plasma tyrosine level (normal range 35–116 µmol/L)

*novel.

**Patient 1** is a 20-year-old man. Dark urine was noted at 13 years of age. His porphyria test was negative, though urine organic acid analysis revealed a large amount of HGA. No arthralgia or hyperpigmentation was present currently.

**Fig. 1:**
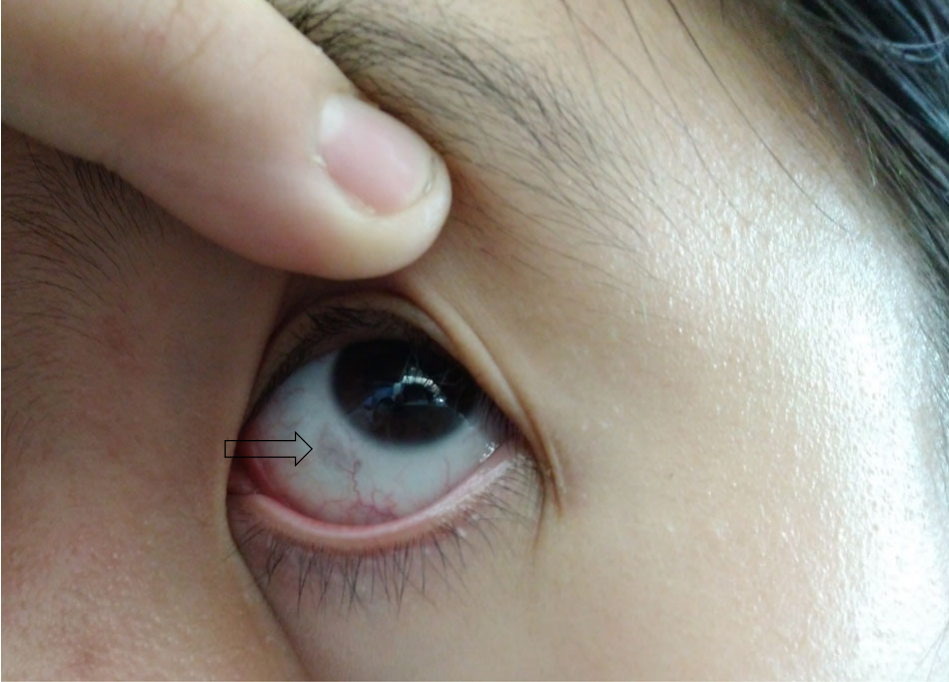
Ocular ochronosis of Patient 2. The lesion on the sclera is pointed out by an open arrow. Written consent was obtained for the publication of this photograph.

**Patient 2** is a 12-year-old female who was noted to have dark urine since early childhood. She was diagnosed at 8 years of age after a urine organic acid analysis, which revealed a large amount of HGA. Currently, she has ochronosis of the sclera (Fig. 1, arrow). No urolithiasis was detected by renal sonography, but she has frequent pain over the right hip and both knees.

**Patient 3** is a 10-year-old boy. He was diagnosed at 5 years of age. When left at room temperature, his urine changed to purple-red in color in 1 hour and was brownish in half a day; these changes were more prominent after eating chocolate or seaweed. A urine organic acid analysis revealed a marked elevation in HGA. At present, he has no hyperpigmentation or urolithiasis; however, he did recently complain about intermittent bilateral knee joint pain.

### Molecular findings

Molecular analyses revealed two *HGD* variants in each patient. Two of these variants have been previously reported: c.291 G > A p.(Trp97Ter) and c.473 C > T p.(Pro158Leu). p.(Trp97Ter), a nonsense variant, is reported as pathogenic in ClinVar, and other pathogenic variants at this amino acid p.(Trp97Arg) and p.(Trp97Gly) have also been reported, indicating that it is likely an important site for disease variants^2,9^. The variant p.(Pro158Leu), which occurs at CpG dinucleotides, is thought to be a mutation hot spot^10,11^. The other four variants have not been reported to date: c.16-2063 A > C, p.(Thr196Ile), p.(Gly344AspfsTer25), and p.(Gly362Arg) (Table 2). The frameshift variants p.(Gly344AspfsTer25) are predicted to be pathogenic. The two missense variants p.(Thr196Ile) and p.(Gly362Arg) are located in beta-strands of the HGD protein and have very low allele frequency in normal populations (maximal minor allele frequency of 0.0003 for p.(Thr196Ile); p.(Gly362Arg) was not previously described). Gly362 is proximal to the iron-binding site (residues 335, 341, and 371) of HGD protein. Both of them were predicted to be pathogenic by mCSM and HGDiscovery^12,13^.

**Table 2:** Novel *HGD* gene variants identified in the current study.

Physical position	REF	ALT	Variant	Type	Pathogenicity score*	SpliceAI	ClinVar/dbSNP	ACMG	DB-ID
3:120,396,773	T	G	c.16-2063 A > C p.Tyr5_Ile6insValTer17	Intronic/frameshift	-	donor gain 0.24 (high recall)	−/−	Likely pathogenic (PS3 PM2 PM3 PP4)	AKU_00248
3:120,365,176	G	A	c.587 C > T p.(Thr196Ile)	Missense	9/13	-	−/rs781491692	Likely pathogenic (PM1 PM2 PP3 PP4)	AKU_00249
3:120,352,151	C	-	c.1031delG p.(Gly344AspfsTer25)	Frameshift	-	-	−/−	Pathogenic (PVS1 PM2 PP3 PP4)	AKU_00245
3:120,352,098	C	T	c.1084 G > A p.(Gly362Arg)	Missense	12/13	-	−/−	Likely Pathogenic (PM1 PM2 PM5 PP3 PP4)	AKU_00246

Reference genome: GRCh37.p13, *HGD transcript* NM_000187.4; REF, Reference allele; ALT, Alternative allele; DB-ID: database-ID of HGD mutation database. *Pathogenicity scores (for missense variants): number of tools predicted as damaging or deletions/13 in silico tools include SIFT, Polyphen-2-DVAR, Polyphen2_HDIV, MutationTaster, FATHMM, PROVEAN, MetaSVM, MetaLR, LRT, MutationAssessor, M_CAP, CADD, and fathmmMKL.

**Fig. 2:**
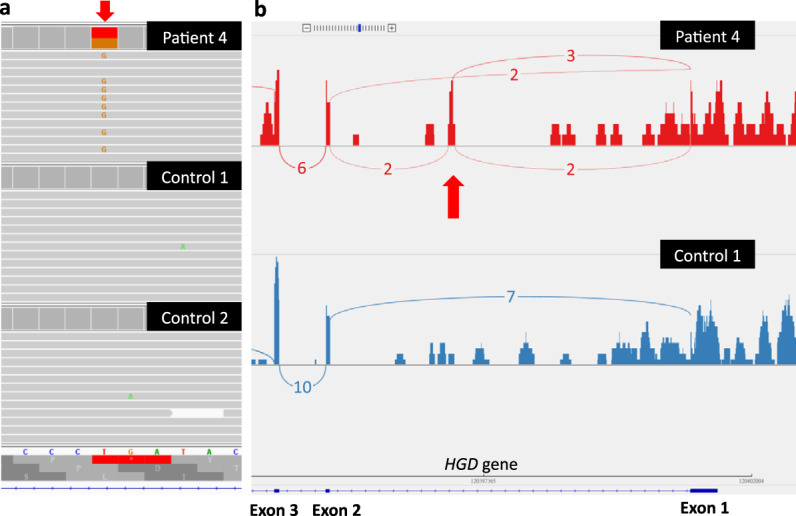
Results of DNA and RNA sequencing. **a** DNA sequencing reveals a heterozygous variant, c.16-2063 A > C (arrow), in Patient 3 but not in the controls. **b** Results from RNA sequencing of Patient 3 and one control. Sashimi plot analysis suggests the inclusion of a cryptic exon (arrow) in the patient. The number of reads supporting the prediction of splicing is marked on the connecting lines.

**Fig. 3:**
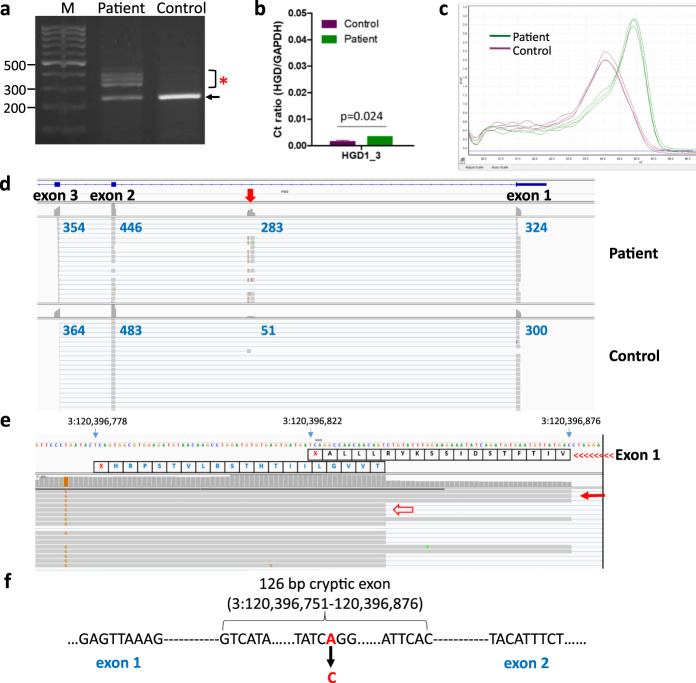
Verification of the effect of the c.16-2063 A > C variant located in intron 1 in Patient 3 by reverse transcription PCR (RT-PCR) of exons 1–3 of *HGD*. **a** Agarose gel electrophoresis analysis reveals, other than the normal product (arrow), several larger-than-expected fragments in the patient (star). **b** Real-time PCR analysis reveals that the total amount of exons 1–3 HGD RNA was higher in Patient 3 than in the control. **c** High-resolution melting analysis reveals a shift in melting temperature in the patient. **d** NGS analysis reveals the inclusion of a 126-bp cryptic exon (283 reads) in half of the products (446 reads for exon 2) in the patient. A small portion of the reads in the control also contains this cryptic exon. There are other less-frequent cryptic exons included in the patient (not shown). **e** A zoom-in view of the cryptic exon reveals a major transcript (arrow) that causes protein truncation (Tyr5_Ile6insValTer17), and a minor transcript that also causes protein truncation (open arrow). **f** The c.16-2063 A > C variant is predicted to disrupt an SRSF5-binding site (TATCAGG) and then activate the inclusion of the cryptic exon (genomic coordinate 3:120,396,751–120,396,876).

DNA sequencing for Patient 3 revealed a heterozygous intronic variant c.16-2063 A > C (Fig. 2a, arrow) with a maximal minor allele frequency of 0.0006. To demonstrate the function of this variant, RNA-sequencing (RNA-Seq) from peripheral blood total RNA of Patient 3 and one control was performed. Sashimi plot analysis of the RNA-Seq data suggested the inclusion of a cryptic exon in the patient (Fig. 2b, arrow). Because the number of reads in RNA-Seq was small, we further verified aberrant splicing by reverse transcription PCR (RT-PCR). The results of RT-PCR of exons 1–3 of *HGD* revealed that, other than the normal product (arrow), several larger-than-expected fragments were found (Fig. 3a, star). Real-time PCR analysis reveals that the total amount of exons 1–3 *HGD* RNA was not decreased in the patient (Fig. 3b), and these PCR products revealed a shift in melting temperature (Fig. 3c). Next-generation sequencing (NGS) analysis of the PCR products revealed the inclusion of a previously described 126-bp cryptic exon in half of the reads in the patient (Fig. 3d). Some reads also contained other less-frequent cryptic exons (data not shown). A small portion of the reads in the control also contained the 126-bp cryptic exon. A zoom-in view of the cryptic exon revealed that the inclusion of the whole cryptic exon would cause frameshift and protein truncation (Fig. 3e). Therefore, variant c.16-2063 A > C is predicted as p.Tyr5_Ile6insValTer17. The c.16-2063 A > C variant is likely to disrupt an SRSF5-binding site (TATCAGG) and then activate the inclusion of the cryptic exon (Fig. 3f).

**Fig. 4:**
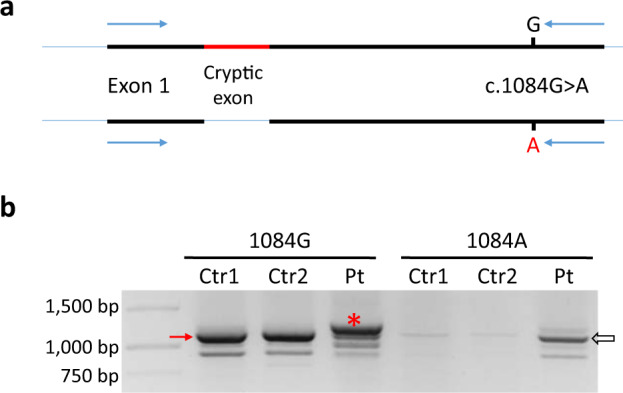
Allele-specific amplification of *HGD* mRNA in Patient 3. **a** Allele-specific PCR was designed for the c.1084 G > A variant. The 1084 A allele is normal in splicing, while the 1084 G allele contains the cryptic exon. The positions of the primers are marked by blue arrows. **b** RT-PCR with the 1084 G wildtype primer gives the 1157-bp products (arrow) in the controls, but gives a major band with a higher molecular weight in the patient. There may be a small amount (18% by densitometry) of normal-size PCR products in the patient. RT-PCR with the 1084 A mutant primer gives the 1157-bp product (open arrow) in the patient but not in the controls.

In order to confirm that the c.16-2063 A > C variant is in trans to another likely pathogenic variant (c.1084 G > A at exon 13), allele-specific PCR was designed for c.1084 G > A (Fig. 4a). The c.16-2063 A > C variant is supposed to be on the 1084 G (wildtype) allele. The results revealed that RT-PCR with the 1084 G primer gave 1157-bp products (Fig. 4b, arrow) in the controls, but gave a major higher molecular weight product (Fig. 4b, star) in the patient. There may be an 1157-bp band in the patient, but that band only constituted 18% of the total PCR product as estimated by densitometry.

### Variant interpretation at the protein level for novel variants

The active form of HGD is a delicate hexamer, and amino-acid residues involving protomer folding, hexamer assembly, and substrate binding have been shown to play important roles in HGD disease variants^4,12^. We employed structure-based variant characterization tools to identify the molecular consequences of the novel variants in the current study, including mCSM, mCSM-lig, mCSM-PPI2, and HGDiscovery (Table 3)^12–19^.

**Table 3:** Novel missense variants predictions.

Predicted protein change	mCSM-stability	Distance to interface	mCSM-PPI2	Distance to ligand	mCSM-Lig	Category of missense variant*	HGDiscovery Prediction
p.(Thr196Ile)	Destabilizing (−0.48 kcal/mol)	23.2 Å	Decreased affinity (−0.41 kcal/mol)	24.8 Å	Decreased affinity (−1.94 log)	Protomer destabilization	Pathogenic
p.(Gly362Arg)	Destabilizing (−0.17 kcal/mol)	14.9 Å	Decreased affinity (−0.59 kcal/mol)	10.3 Å	Decreased affinity (−1.27 log)	Protomer destabilization, hexamer disruption, and active site disruption	Pathogenic

*The classes are not necessarily mutually exclusive. The largest effect of both variants is on the stability of the protomer; but Gly362Arg is also likely to disrupt the formation of the hexamer and ligand binding.

**Fig. 5:**
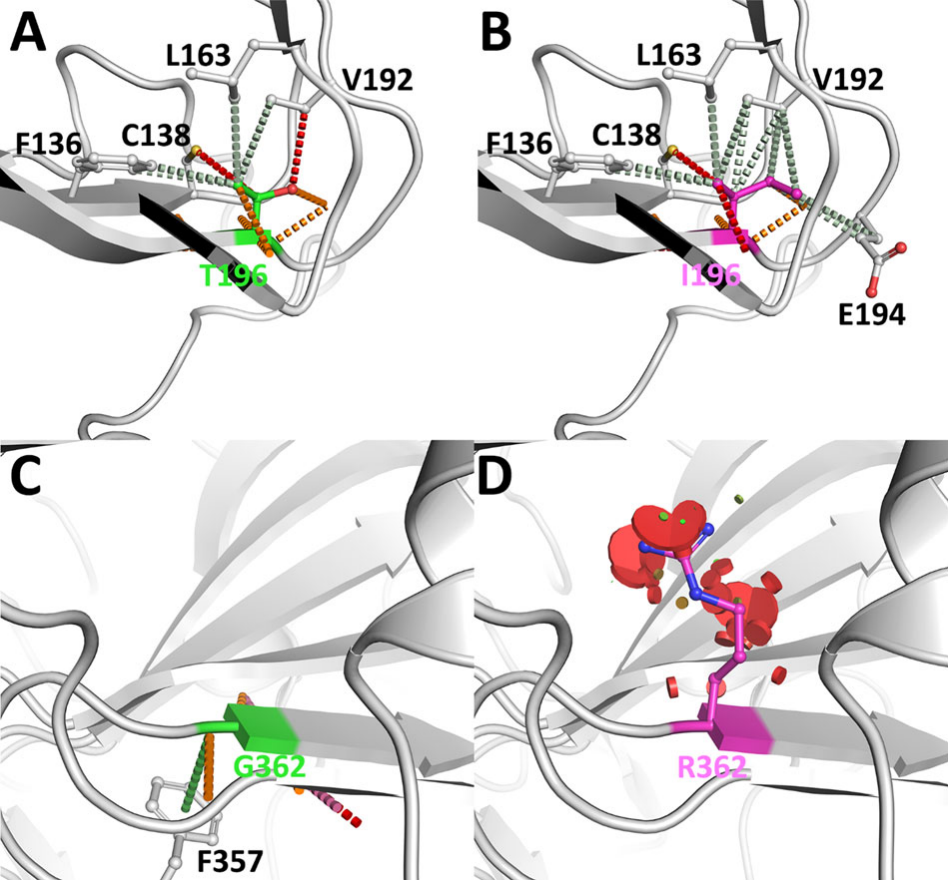
Intramolecular interactions of novel HGD missense variants calculated using Arpeggio^28^. The sidechain of Thr196 (**A**) makes a series of hydrogen bonds to neighboring residues, which would be lost upon variant to Ile (**B**). A variant of the positive phi Gly362 (**C**) to Arg (**D**) would lead to large steric clashes within the structure of HGD. This highlights that both variants would lead to significant structural consequences and be quite deleterious. The wild-type residues are shown as green sticks, and the mutant residue is in magenta sticks. Hydrogen bonds are shown as red dashed lines, polar interactions as orange dashed lines, hydrophobic interactions as green dashed lines, and steric clashes as red disks.

Thr196 is located distal to the active site and hexamer interface (Fig. 5A). Therefore, its variant to Ile is unlikely to disrupt substrate binding or the formation of the active hexamer. Thr196 is, however, a buried and conserved residue that is intolerant to missense variant, and the introduction of an Ile leads to the loss of key hydrogen bonds to Val198 and mild steric clashes (Fig. 5B). Consistent with this, the variants of Thr196Ile are predicted to mildly destabilize the HGD structure, leading to it being predicted by HGDiscovery as pathogenic.

Gly362 is a buried residue located 10 Å from the active site and within 15 Å of the hexamer interface (Fig. 5C). As a positive phi glycine, variant to arginine is likely to lead to significant disruption of the protein structure. The introduction of the larger arginine sidechain is also harder to accommodate within the tightly packed buried core and leads to significant steric clashes (Fig. 5D). This is consistent with G364 being identified as intolerant to missense variants and predicted to destabilize the protein, hexameric structure, and ligand binding. It was consequently identified as pathogenic by HGDiscovery.

## Discussion

AKU has never been reported in Taiwan before, and we observed only three cases in a medical center that treats the largest number of rare disorders in Taiwan. Usually, symptoms of AKU initiate from ochronotic pigmentation in the 4th decade of life, which is followed by painful joint destruction requiring artificial joint replacement and cardiac valvular problems in the mid-50s and the formation of renal stones in the 60 s^2^. However, some of our three patients as young as 12 years already had ochronosis or arthralgia. Nevertheless, none of the 10- and 12-year-olds with arthralgia presented with joint destruction. Therefore, it is possible that the joint pain was psychological owing to the stress from having a chronic illness.

Biochemical diagnosis of AKU based on the detection of HGA in urine samples is accurate. Nevertheless, although HGA can be detected in routine urinary organic acid analysis, the disease is so rare that the operator may not recognize the compound unless they are reminded by a physician who suspects this diagnosis. Molecular diagnosis of AKU can also be challenging, especially in East Asia, where the disease is extremely rare and most of the variants are novel. For example, the deep intron variant c.16-2063 A > C detected in Taiwanese patients are not included in any of the human genome databases. Therefore, we needed to perform additional analyses to demonstrate the pathogenicity of this variant that affects splicing. It was fortunate that the cryptic exon has been described, thus, our capture probes included this exon; otherwise, we would not have detected this variant. RNA-Seq is certainly very helpful for revealing splicing aberrations. MetaDome software is also useful because this software provides good visualization of the tolerability of variants over the entire protein. With this tool, it is easier to evaluate novel variants by comparing them with other reported variants, which is especially helpful in ultrarare diseases such as AKU.

**Fig. 6:**
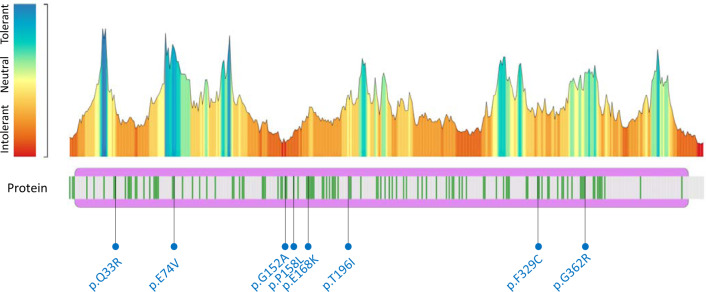
Pathogenicity analysis of known *HGD* missense variants by MetaDome. Green lines indicate all variants reported by HGMD and ClinVar. Round dots indicate variants reported in East Asia.

We retrieved 100 articles from PubMed but excluded 76 of them due to lack of information; we added both HGMD and ClinVar records. Not including our cohort, a total of 223 variants in 594 patients have been reported as disease-causing variants at the time of calculation (Dec 2020). However, only 6 patients from East Asia (China, South Korea, and Japan) are reported in PubMed^20–25^. Together with our three patients, 13 of the 18 East Asian mutated alleles have not been detected in other countries. The 13 mutated alleles comprise 12 variants p.(Gln33 Arg), p.(Glu42SerfsTer69), p.(Glu74Val), c.342 + 3A > C, p.(Gly152Ala), p.(Thr196Ile), p.(Glu329Cys), p.(Gly344AspfsTer25), p.(Gly362Arg), p.(Ser366_Thr367dup), c.16-2063 A > C, and c.469 + 1 G > C. Therefore, most of the AKU patients in East Asia harbor private variants in *HGD*. All *HGD* missense variants were analyzed by the pathogenicity analysis tool MetaDome, and the results revealed that most are located in intolerant parts of the protein; for the Asian variants, tolerant for p.(Glu74Val), slightly tolerant for p.(Gly362Arg), neutral for p.(Thr196Ile), slightly intolerant for p.(Gln33Arg) and p.(Phe329Cys), intolerant for p.(Pro158Leu) and p.(Glu168Lys), and highly intolerant for p.(Gly152Ala) (Fig. 6).

We only identified three cases in our institute, and there is no previous report of AKU in Taiwan. Although we were not able to calculate the prevalence of AKU in Taiwan, the disease must be very rare, as in all of East Asia. In addition, we were unable to fully elucidate the phenotype of the disease in our four Taiwanese patients because they were still young. Nonetheless, the suspicious ochronosis and arthralgia in a portion of them alert us to the potential for more serious symptoms in the future.

Alkaptonuria is rare in Taiwan and East Asia, with *HGD* variants being mostly novel. More efforts involving molecular analysis are expected to contribute to the diagnosis of new cases in this region.

## Materials and methods

### Patients

From Jan 2010 to Jun 2020, patients diagnosed with AKU at National Taiwan University Hospital were reviewed. Informed consent was obtained from all subjects or their guardians, and the guardian of patient 2 provided written consent for the publication of the photograph in Fig. 1. Clinical information, including the age of onset and clinical presentation, was collected. Diagnostic biochemical analyses performed for these patients included urine organic acid analysis by gas chromatography-mass spectrometry or thin-layer chromatography to reveal the presence of HGA in urine samples. The study was approved by the Institutional Review Board (IRB No. 201505135RIN) in our hospital.

### Molecular analyses

Variant analyses of *HGD* (RefSeq: NM_000187.4) were performed by targeted panel sequencing using a SeqCap EZ probe (Roche Nimbelgen, Basel, Switzerland) and MiSeq sequencer (Illumina, San Diego, CA, USA) to produce 300-bp paired-end reads, with an average coverage >150 crossing exons and flanking intronic (50 bp) sequences. Sequence alignment to the human reference genome (GRCh37) was performed using Burrows-Wheeler Aligner, and variant calling was performed using Genome Analysis Tool Kit (GATK v4.0, Broad Institute)^26^. Variants were annotated by ANNOVAR (http://wannovar.wglab.org/)^27^. DNA and protein sequence variants were described as recommended by Human Genome Variation Society (HGVS, http://varnomen.hgvs.org/). For missense variants, we employed Sorting Intolerant From Tolerant (SIFT, https://sift.bii.a-star.edu.sg/), PolyPhen-2 (Polymorphism Phenotyping v2, http://genetics.bwh.harvard.edu/pph2/), mCSM (http://biosig.unimelb.edu.au/mcsm/)^16^, mCSM-PPI2 (http://biosig.unimelb.edu.au/mcsm_ppi2/)^15,18^ and mCSM-lig (http://biosig.unimelb.edu.au/mcsm_lig/)^17^ to predict effects on protein function and structure using the experimental crystal structure of HGD (PDB ID: 1EY2). Intramolecular interactions were calculated and visualized using Arpeggio^28^ and mutational tolerance was calculated using MTR-Viewer^13,19^. For intronic variants, we used Human Splicing Finder (http://umd.be/Redirect.html) and Splice AI to predict their splice effects. We also searched the *HGD* mutation database (http://hgddatabase.cvtisr.sk/home.php) and ClinVar to assess whether variants have been reported. The pathogenicity of variants was classified according to the American College of Medical Genetics and Genomics (ACMG) and the Association for Molecular Pathology guidelines^29^.

RNA-Seq was performed on total RNA extracted from peripheral whole-blood samples. After the depletion of ribosomal RNA and globin mRNA, each sample was sequenced with a total output of 50 M reads. Data obtained from RNA-Seq were aligned using STAR and annotated for DNA analysis. RT-PCR was further applied to validate aberrant splicing. The region of exon 1–3 was amplified to demonstrate aberrant splicing. For quantitative PCR using the SYBR Master Mix (Applied Biosystems), *GAPDH* was used as an internal control. HRM was performed by Rotor-gene Q (Qiagen). Allele-specific PCR for RNA was designed for the c.1084 G > A variant, and the specific lower primers were 1084 G (wildtype) 5’-tcattgtgctgtgtagactccc and 1084 A (mutant) 5’-tcattgtgctgtgtagactcct. Densitometry was performed using ImageJ. All primer sequences are listed in Supplementary Table 1. All blots derived from the same experiment and were processed in parallel.

Computational analysis to evaluate possible enzyme inactivation of novel missense variants of HGD was used by webserver of HGDiscovery (http://biosig.unimelb.edu.au/hgdiscovery/submit_prediction)^12,13,15^. Asian *HGD* variants were illustrated by the tolerance landscape using Metadome Version 1.0.1 webserver (https://stuart.radboudumc.nl/metadome/dashboard)^14^.

### Literature search

We searched PubMed publications using the criteria “(alkaptonuria) AND ((mutation) OR (variant) OR (gene diagnosis))”. We targeted studies published after 1996, and the time the human *HGD* gene was mapped to chromosome 3q. Exclusion criteria were as follows: 1. cases without ethnicity mentioned or a clear genotype, 2. literature without full-text availability, and 3. literature not written in English or Chinese. We then checked ClinVar, Human Gene Mutation Database (HGMD), and the *HGD* mutation database from Leiden Open Variation Database (LOVD) for the *HGD* mutations described in the publications retrieved.

### Reporting summary

Further information on research design is available in the Nature Research Reporting Summary linked to this article.

## Supplementary information

Supplementary Information

Reporting Summary

## Data Availability

Sequence data have been deposited at the NCBI (SRA), under accession number PRJNA765906. Variants are available in *HGD* mutation database (http://hgddatabase.cvtisr.sk/home.php) under number AKU_00248, AKU_00249, AKU_00245, and AKU_00246. All other data that support the findings of this study are available from the corresponding author upon request.
