# Supplementary material for: A novel deep intronic variant strongly associates with Alkaptonuria
Source: NPJ Genom Med. 2021 Oct 22;6:89. doi: 10.1038/s41525-021-00252-2 (PMC8536767; doi:10.1038/s41525-021-00252-2)

Supplementary Table 1. Primer sequences used in this study

**Item**	**Forward**	**Reverse**	**Size (bp)**
Fig3a- RT-PCR	5’-CATTGTGGCTTTATATCCTCTGG-3’	5’-TACAGCCAGCTTCTCTTATTGGT-3’	250
Fig3b- Real-time PCR	5’-AGGAGAGAGAAAATGGCTGAGT-3’	5’-GGACAAGTGAAAGCCGATCC-3’	166
Fig4- AS-RT-PCR for G1084G	5’-TATATCCTCTGGGCCTGGGT-3’	5’-TCATTGTGCTGTGTAGACTCCC-3’	1157
Fig4- AS-RT-PCR for G1084A	5’-TATATCCTCTGGGCCTGGGT-3’	5’-TCATTGTGCTGTGTAGACTCCT-3’	1157

AS-RT-PCR: allele-specific reverse-transcription PCR; RT-PCR: reverse-transcription PCR

Supplementary figure 1. Un-cropped image of fig. 3a

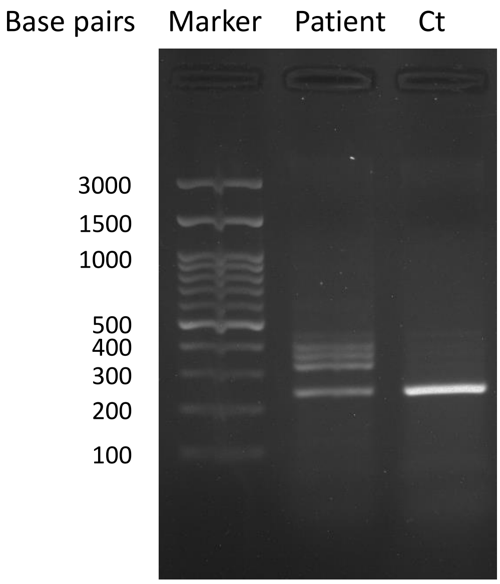

Supplementary figure 2. Un-cropped image of fig. 4b

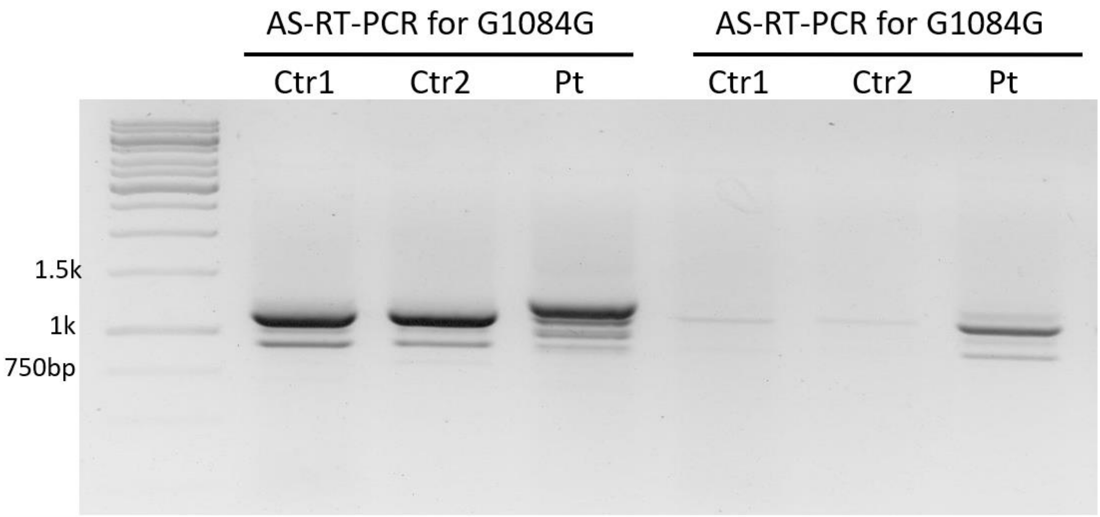

Supplement: Supplementary file 1 — Supplementary Information [file 41525_2021_252_MOESM1_ESM.pdf]
